# Two Subpopulations of Noradrenergic Neurons in the Locus Coeruleus Complex Distinguished by Expression of the Dorsal Neural Tube Marker *Pax7*

**DOI:** 10.3389/fnana.2017.00060

**Published:** 2017-07-20

**Authors:** Nicholas W. Plummer, Erica L. Scappini, Kathleen G. Smith, Charles J. Tucker, Patricia Jensen

**Affiliations:** ^1^Neurobiology Laboratory, National Institute of Environmental Health Sciences, National Institutes of Health, United States Department of Health and Human Services Durham, NC, United States; ^2^Signal Transduction Laboratory, National Institute of Environmental Health Sciences, National Institutes of Health, United States Department of Health and Human Services Durham, NC, United States

**Keywords:** intersectional fate mapping, recombinases, locus coeruleus, norepinephrine, *Pax7*, *En1*, *Dbh*, rhombomeres

## Abstract

Central noradrenergic neurons, collectively defined by synthesis of the neurotransmitter norepinephrine, are a diverse collection of cells in the hindbrain, differing in their anatomy, physiological and behavioral functions, and susceptibility to disease and environmental insult. To investigate the developmental basis of this heterogeneity, we have used an intersectional genetic fate mapping strategy in mice to study the dorsoventral origins of the *En1*-derived locus coeruleus (LC) complex which encompasses virtually all of the anatomically defined LC proper, as well as a portion of the A7 and subcoeruleus (SubC) noradrenergic nuclei. We show that the noradrenergic neurons of the LC complex originate in two different territories of the *En1* expression domain in the embryonic hindbrain. Consistent with prior studies, we confirm that the majority of the LC proper arises from the alar plate, the dorsal domain of the neural tube, as defined by expression of *Pax7*^*Cre*^. In addition, our analysis shows that a large proportion of the *En1*-derived A7 and SubC nuclei also originate in the *Pax7*^*Cre*^-defined alar plate. Surprisingly, however, we identify a smaller subpopulation of the LC complex that arises from outside the *Pax7*^*Cre*^ expression domain. We characterize the distribution of these neurons within the LC complex, their cell morphology, and their axonal projection pattern. Compared to the broader LC complex, the newly identified *Pax7*^*Cre*^-negative noradrenergic subpopulation has very sparse projections to thalamic nuclei, suggestive of distinct functions. This developmental genetic analysis opens new avenues of investigation into the functional diversity of the LC complex.

## Introduction

Collectively defined by their synthesis of the neurotransmitter norepinephrine, central noradrenergic neurons are heterogeneous. In addition to varying in their anatomical location, morphology, axonal projection pattern, electrophysiological characteristics, and gene expression (Dahlström and Fuxe, 1964; Swanson, 1976; Holets et al., 1988; Robertson et al., 2013; Chandler et al., 2014; Li et al., 2016), noradrenergic neurons exhibit differential susceptibility to neurodegenerative disease and toxicant exposure (German et al., 1992; Mohideen et al., 2011; Theofilas et al., 2017). Determining the molecular basis of this heterogeneity is therefore likely to have important clinical implications, in addition to shedding light on how noradrenergic neurons regulate behaviors and physiological functions as diverse as attention, emotion, appetite, memory, and response to stress (Berridge and Waterhouse, 2003).

The organization of central noradrenergic neurons was initially defined over fifty years ago by their rostrocaudal position in the adult brainstem (Dahlström and Fuxe, 1964). This mature anatomical organization is still the primary means by which the noradrenergic system is studied, but evidence of heterogeneity within anatomically defined noradrenergic nuclei indicates that other approaches will be required to fully explain functional diversity. An alternative strategy for understanding this heterogeneity is the study of genetic neuroanatomy, correlating gene expression with brain structure and function (Joyner and Sudarov, 2012). To this end, we have recently used a recombinase-mediated intersectional genetic fate mapping approach to subdivide the mature noradrenergic system based on gene expression differences along the anteroposterior axis of the embryonic hindbrain (Robertson et al., 2013, 2016). We identified genetically defined noradrenergic subpopulations originating in different rhombomeres (r), transient segments of the embryonic hindbrain with distinct gene expression profiles (Krumlauf et al., 1993; Chambers et al., 2009). The population arising from the isthmus (r0) and r1, as defined by early expression of *En1*, comprises the majority of the locus coeruleus (LC), the largest anatomically defined noradrenergic population and a major source of norepinephrine released in the cortex, as well as part of the dorsal subcoeruleus (SubC) and A7 noradrenergic nuclei in the pons. More recently, fate mapping on the basis of *Fgf8*^*Cre*^ expression in r0 has demonstrated that the LC is divided between r0- and r1-derived subpopulations (Watson et al., 2017). A dorsal subpopulation is derived from the *Fgf8*^*Cre*^ expression domain encompassing r0, and by inference, the remaining ventral subpopulation is derived from r1.

In addition to the anteroposterior axis, developmental gene expression in the embryonic neural tube also varies on the dorsoventral axis (Echelard et al., 1993; Liem et al., 1995, 1997; Moreno-Bravo et al., 2014; Di Bonito and Studer, 2017). This second axis of developmental gene expression is likely a source of functional heterogeneity in the mature LC and adjacent noradrenergic neuron populations. The developmental progression of *Phox2a* and *Phox2b* expression during LC development suggests that the neurons of the LC originate in the alar plate, the dorsal subdivision of the neural tube, and migrate to their final position in the basal plate (Aroca et al., 2006). In chick, the alar origin of the LC was confirmed experimentally by fate mapping with quail/chick chimeras and immunohistochemical analysis using *Pax7* as a marker of the alar plate (Aroca et al., 2006). The dorsoventral origins of SubC and A7 noradrenergic neurons derived from the *En1* expression domain were not assessed.

In the current study, we use an intersectional genetic fate mapping strategy in mice to directly assess the dorsoventral origins of all the noradrenergic neurons derived from the *En1* expression domain encompassing r0 and r1. We confirm that the majority of LC neurons originate in the alar plate, as defined by expression of *Pax7*^*Cre*^, and demonstrate that this region is also the source of a large proportion of r0- and r1-derived SubC and A7 cells. Surprisingly, we also find that a small subpopulation of noradrenergic neurons distributed throughout the mature LC, SubC, and A7 does not have a history of *Pax7*^*Cre*^ expression, indicating an origin outside the *Pax7*-defined alar plate. Consistent with the hypothesis that different developmental origins imply different functions, this subpopulation has a distinct axonal projection pattern.

## Materials and methods

### Animals

This study was performed in accordance with the recommendations in the Guide for the Care and Use of Laboratory animals of the National Institutes of Health. The protocols were approved by the Animal Care and Use Committee (ACUC) of the National Institute of Environmental Health Sciences.

All mouse lines used in this study are listed in Table 1 and were maintained by back-crossing to C57BL/6J mice. To generate the new *RC::LTG* fluorescent indicator line, *RC::RFLTG* mice were crossed with the *CAG-dre* and *ACT-Flpe* recombinase driver lines to permanently remove the rox- and FRT-flanked transcriptional stop cassettes. To label single-recombinase expression domains in embryos, we crossed *En1*^*Dre*^ with *RC::RLTG* mice, and *Pax7*^*Cre*^ with *RC::LTG* mice. To label subpopulations of noradrenergic neurons derived from r0 and r1, *RC::RFLTG* mice were intercrossed with *En1*^*Dre*^, *Pax7*^*Cre*^, and *Dbh*^*Flpo*^, generating mice heterozygous for *RC::RFLTG* and all three recombinase driver alleles. Littermates heterozygous for one or two recombinase drivers served as controls.

**Table 1:** Mouse lines used in this study.

**Abbreviated nomenclature**	**Standardized genetic nomenclature**	**Repository stock no**.	**References**
*RC::RFLTG*	B6.Cg-*Gt(ROSA)26Sor^*tm*1.1(*CAG*−*tdTomato*, −*EGFP*)*Pjen*^*	JAX 026930	Plummer et al., 2015
*RC::RLTG*	B6.Cg-*Gt(ROSA)26Sor^*tm*1.2(*CAG*−*tdTomato*, −*EGFP*)*Pjen*^*	JAX 026931	Plummer et al., 2015
*RC::LTG*	B6.Cg-*Gt(ROSA)26Sor^*tm*1.4(*CAG*−*tdTomato*, −*EGFP*)*Pjen*^*		this manuscript
*En1^*Dre*^*	B6.129-*En1^*tm*1.1(*dreo*)*Pjen*^*		Plummer et al., 2016
*Dbh^*Flpo*^*	B6;129-*Dbh^*tm*1(*flpo*)*Pjen*^*		Robertson et al., 2013
*Pax7^*Cre*^*	STOCK *Pax7^*tm*1(*cre*)*Mrc*^*/J	JAX 010530	Keller et al., 2004
*CAG-dre*	B6;129-*Tg(CAG-dre)1Afst*	MMRRC 032246-UCD	Anastassiadis et al., 2009
*ACT-Flpe*	B6.Cg-*Tg(ACTFlpe)9205Dym*/J	JAX 005703	Rodriguez et al., 2000

### Tissue collection

For timed matings, noon of the day on which mating plugs were identified was taken to be embryonic day (E) 0.5. Embryos were fixed by immersion in 4% paraformaldehyde (PFA) in 0.01 M phosphate buffered saline (PBS) at 4°C overnight. Following equilibration in 10, 20, and 30% sucrose in PBS, embryos were embedded in Tissue Freezing Medium (General Data Company, Cincinnati, OH), and 14-μm cryosections were mounted on Superfrost Plus microscope slides (Thermo Scientific, Waltham, MA), air dried and stored at −80°C.

For collection of adult brain tissue, mice were anesthetized with sodium pentobarbital and transcardially perfused with 4% PFA. After dissection, brains were post-fixed by immersion in 4% PFA at 4°C overnight. Following rinse with PBS, brains to be processed for immunohistochemistry were equilibrated in 30% sucrose for 48 h at 4°C, and embedded in Tissue Freezing medium. Free-floating 40-μm cryosections were collected and stored at −80°C in 30% sucrose/30% ethylene glycol in PBS. Brains to be cleared by the passive clarity technique were processed according to the protocol below.

### Immunohistochemistry

For immunofluorescent labeling, EGFP-expressing cells in slide-mounted embryo sections and free-floating adult brain sections were detected with chicken anti-GFP primary antibody (1:10,000; Cat.# ab13970, Abcam, Campridge, MA) and Alexa Fluor 488 goat anti-chicken secondary antibody (1:1,000; Cat.# A11039, Life Technologies, Grand Island, NY). tdTomato-expressing cells were detected with rabbit anti-dsRed primary antibody (1:1,000; Cat.#632496, Clontech Laboratories, Mountain View, CA) and Alexa Fluor 568 goat anti-rabbit secondary antibody (1:1,000; Cat.# A11036, Life Technologies). Noradrenergic neuron cell bodies were detected with mouse monoclonal anti-tyrosine hydroxylase (1:500; clone 185, Cat.# GTX10372, GeneTex, Irvine, CA) and Alexa Fluor 633 goat anti-mouse secondary antibody (1:1,000; Cat.# A21052, Life Technologies). After immunolabeling, adult brain sections were mounted on Superfrost Plus slides. Both embryo and adult sections were incubated with TrueBlack reagent (Biotium, Fremont, CA) to quench autofluorescence, and coverslipped with VectaShield plus DAPI (4′,6-diamidino-2-phenylindol) Hard Set mounting medium (Vector Laboratories, Burlingame, CA).

For highest sensitivity detection of EGFP and tdTomato-expressing axons, we used immunoperoxidase labeling of 40-μm free-floating sections (*n* = 8 mice). The chicken anti-GFP antibody (1:10,000) was used in conjunction with a biotinylated goat anti-chicken secondary antibody (1:500; Cat.# BA-9010, Vector Laboratories), and rabbit anti-dsRed (1:1,000) was used with biotinylated goat anti-rabbit secondary antibody (1:500; Cat.# BA-1000; Vector Laboratories). Immunoreactivity was detected with the Vectastain Elite ABC kit and DAB Peroxidase Substrate Kit (both Vector Laboratories), and slides were coverslipped with Permount mounting medium (Fisher Scientific, Waltham, MA).

### Passive clarity tissue clearing

The passive clarity technique (PACT) (Yang et al., 2014) and immunofluorescent labeling of cleared tissue were performed as previously described (Plummer et al., 2015) except for the following modifications. Following the fixation protocol described above, 2 or 3 mm-thick coronal brain slices were embedded in 4% polyacrylamide gel (A4P0). Lipids were extracted by incubation in 8% SDS in PBS at 37°C for 6 days. During the incubation, the SDS was replaced with fresh solution every other day. For immunohistochemistry, tissue was incubated with chicken anti-GFP (1:1,000; Cat.# ab13970, Abcam) and rabbit anti-dsRed (1:500; Cat.#632496, Clontech Laboratories) primary antibodies, followed by incubation with Alexa Fluor 488 donkey anti-chicken F(ab')2 fragments (1:500; Cat.# 703-546-155, Jackson ImmunoResearch Laboratories, West Grove, PA) and Alexa Fluor 568 donkey anti-rabbit F(ab')2 fragments (1:500; Cat.# Ab175694, Abcam), for 6 days each, with buffer and antibody replaced with fresh solution after 3 days. To inhibit bacterial growth, 0.03% sodium azide was included in all antibody solutions, not 0.01% as previously published (Plummer et al., 2015).

### Digital image collection and processing

Tile scan images of fluorescently labeled sections were collected on a Zeiss LSM 780 or 880 inverted confocal microscope (Carl Zeiss Microscopy, Thornwood, NY), and z-stacks were converted to maximum intensity projections using Zen 2012 Black Software (Carl Zeiss). Images were modified only by using Photoshop (Adobe Systems, San Jose, CA) or ImageJ software (US National Institutes of Health) to adjust brightness and contrast across the entire image. Tile scan images of tissue cleared by PACT were collected on an LSM 880 confocal microscope using an EC Plan-Neofluar 10x/0.3 M27 objective (Carl Zeiss). Due to the thickness of the samples, the Auto Z Brightness Correction was used for both the 488 nm laser line from an Argon laser (2–20% power range) and 561 nm laser line from a DPSS laser (2–12% power range). The pinhole was set to yield an optical z-thickness of 14 μm, and a z-stack was collected at 10 μm interval between images. z-stacks were viewed with Imaris Software (Bitplane, Concord MA) for three-dimensional rendering of the entire region and surface rendering of individual cells.

### Cell counts

EGFP- and tdTomato-labeled neurons were counted in the z-stacks encompassing the entire population of r0- and r1-derived noradrenergic neurons in PACT cleared brain tissue from *En1*^*Dre*^*; Dbh*^*Flpo*^*; Pax7*^*Cre*^*; RC::RFLTG* mice (*n* = 6). Images were cropped, and a subset consisting of a z-slice every 60 μm (the thickness of a cell body on the z axis in the cleared tissue) was made in Zen 2012 Black Software (Carl Zeiss). We compared several adjacent z-slices with the full z-stack to confirm that this separation distance does not result in missed neurons or double counting of the same neuron in adjacent z-slices. The individual images were then imported into FIJI software (Schindelin et al., 2012) for smoothing and application of the eliminate maxima filter from the Fast Filters plugin. Labeled neurons were counted using the Cell Counter plugin in FIJI. Cell counts are reported as mean ± standard error. Cell numbers in left and right hemispheres were compared by unpaired *t*-test using Graphpad Prism software (Graphpad, La Jolla, CA).

## Results

Using intersectional genetic fate-mapping, we have previously shown that noradrenergic neurons derived from the *En1* expression domain of the embryonic hindbrain populate 99.8% of the locus coeruleus (LC) and a portion of the dorsal subcoeruleus and A7 nuclei (Robertson et al., 2013) according to an adult mouse brain atlas (Paxinos and Franklin, 2013). These neurons (hereafter designated the LC complex) form a continuum extending from the compact LC proper to the more dispersed cells of the SubC and A7 nuclei (Plummer et al., 2015). An earlier study using *in situ* hybridization of chick and mouse embryos and quail/chick grafting experiments demonstrated that the LC is derived from the alar plate (Aroca et al., 2006), but these embryonic analyses did not distinguish the LC proper from the SubC and A7, structures more clearly delineated in the adult brain. To determine if all noradrenergic neurons of the LC complex originate from the alar plate, we therefore employed a recombinase-based intersectional and subtractive genetic fate mapping strategy that allows labeled neurons to be mapped into the adult brain (Jensen and Dymecki, 2014).

**Figure 1:**
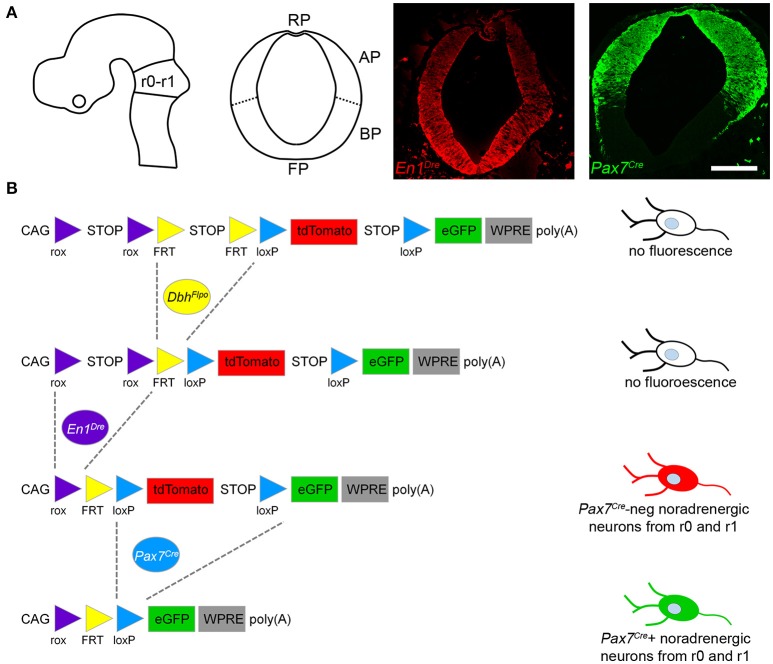
Recombinase driver lines expressed in the embryonic hindbrain permit intersectional genetic fate mapping of noradrenergic neuron subpopulations. **(A)** Schematic diagrams show the mouse neural tube at E10.5 (Left, sagittal view; Right, coronal view) at the level of rhombomeres 0 and 1 (r0-r1). In E10.5 embryos a fluorescent Dre indicator reveals *En1*^*Dre*^ expression throughout the dorsoventral axis, while a fluorescent Cre indicator shows *Pax7*^*Cre*^ restricted to the alar plate (AP). BP, Basal plate; FP, floor plate; RP, roof plate. Scale bar: 200 μm. **(B)** Schematic diagram of the *RC::RFLTG* indicator allele (Plummer et al., 2015). *En1*^*Dre*^ and *Dbh*^*Flpo*^ recombinase activity deletes rox- and FRT-flanked stop cassettes, permitting tdTomato expression in noradrenergic neurons originating in r0 and r1 outside the *Pax7*^*Cre*^ domain. *En1*^*Dre*^, *Dbh*^*Flpo*^, and *Pax7*^*Cre*^ expression deletes the stop cassettes and tdTomato, permitting EGFP expression in noradrenergic neurons from the *Pax7*^*Cre*^ domain of r0 and r1. Schematic does not represent chronological order of recombinase expression.

Mapping noradrenergic neurons derived from the alar plate of r0 and r1 required three recombinase driver alleles and a triple recombinase-responsive indicator allele. *Dbh*^*Flpo*^ (Robertson et al., 2013) is a noradrenergic-specific Flp recombinase driver controlled by the promoter of dopamine β-hydroxylase (*Dbh*), the enzyme required for the final step in norepinephrine synthesis. *En1*^*Dre*^ (Plummer et al., 2016) expresses Dre recombinase in the embryonic midbrain, r0, and r1 (Figure 1A), and *Pax7*^*Cre*^ (Keller et al., 2004) expresses Cre in the alar plate (Figure 1A). The indicator allele, *RC::RFLTG* (Plummer et al., 2015), expresses tdTomato after Dre-mediated deletion of a rox-flanked transcriptional stop cassette and Flp-mediated deletion of a FRT-flanked stop cassette. EGFP is expressed following Dre-, Flp-, and Cre-mediated deletion of all stop cassettes and the loxP-flanked tdTomato.

In mice heterozygous for all three recombinase driver alleles and the indicator allele, noradrenergic neurons derived from the *Pax7*^*Cre*^-defined alar plate of r0 and r1 (the intersectional population) will be selectively labeled with EGFP (Figure 1B). Any noradrenergic neurons derived from r0 and r1 outside of the *Pax7*^*Cre*^ domain (the subtractive population) will be labeled with tdTomato, and noradrenergic neurons originating outside of r0 and r1 will not be labeled due to the presence of the rox-flanked stop cassette (Figure 1B). Co-labeling of the same neuron with EGFP and tdTomato is not possible, because of the order in which driver expression occurs. *Pax7*^*Cre*^ activity can be detected at E10.5 (Figure 1A), prior to the detection of *Dbh*^*Flpo*^ activity (Robertson et al., 2013). Therefore, *Pax7*^*Cre*^-mediated recombination will delete the tdTomato cassette in progenitor cells before *Dbh*^*Flpo*^-mediated recombination in postmitotic noradrenergic neurons permits fluorescent protein expression.

**Figure 2:**
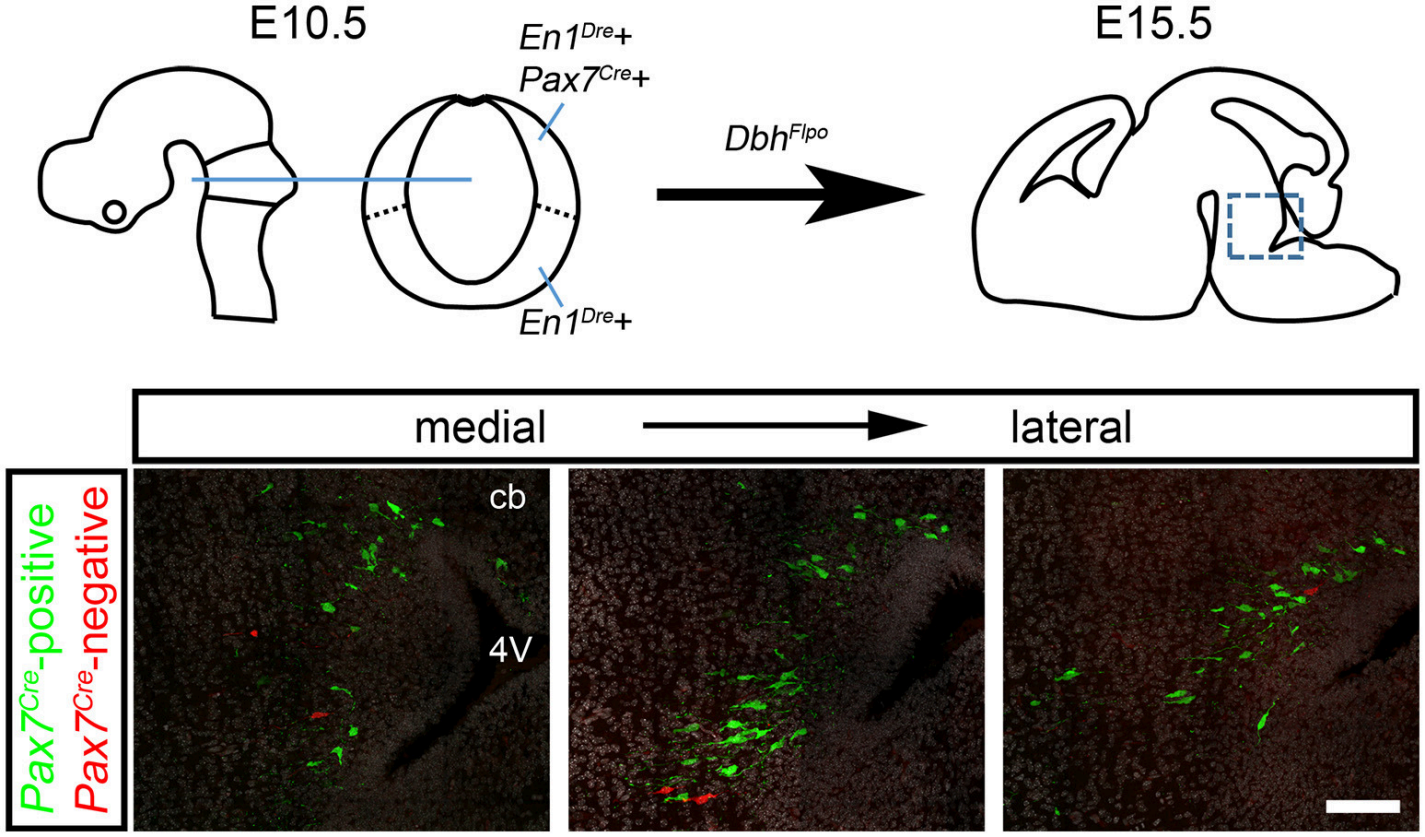
A subpopulation of r0- and r1-derived noradrenergic neurons does not come from the *Pax7*^*Cre*^-defined alar plate. Schematic diagrams show the mouse brain at E10.5 and E15.5. On the sagittal schematic of E15.5 brain, the box indicates the approximate position of immunofluorescent images shown below. In fluorescently labeled parasagittal sections of an E15.5 *En1*^*Dre*^*; Dbh*^*Flpo*^*; Pax7*^*Cre*^*; RC::RFLTG* quadruple heterozygous embryo, the majority of r0- and r1-derived noradrenergic neurons are labeled with eGFP, indicating a history of *Pax7*^*Cre*^ expression. At several different points along the mediolateral axis, a few neurons are labeled with tdTomato, indicating an origin outside the *Pax7*^*Cre*^-defined alar plate. DAPI staining (false colored gray) reveals tissue that is not otherwise stained. cb, Cerebellum; 4V, fourth ventricle. Scale bar: 100 μm.

In the developing hindbrain of E15.5 embryos heterozygous for all three recombinase drivers and the indicator allele, we observed predominantly EGFP-labeled *Pax7*^*Cre*^-positive noradrenergic neurons (Figure 2), consistent with the prior observation that the locus coeruleus is derived from the alar plate (Aroca et al., 2006). However, we consistently observed a small population of tdTomato-labeled *Pax7*^*Cre*^-negative noradrenergic neurons intermingled with the EGFP-labeled neurons (Figure 2). As expected, in control littermates heterozygous for *En1*^*Dre*^ and *Dbh*^*Flpo*^, but not *Pax7*^*Cre*^, all r0- and r1-derived noradrenergic neurons were labeled with tdTomato, and no labeling was observed in other genotypes. These results indicate that a subpopulation of r0- and r1-derived noradrenergic neurons originates outside the *Pax7*^*Cre*^-defined alar plate.

**Figure 3:**
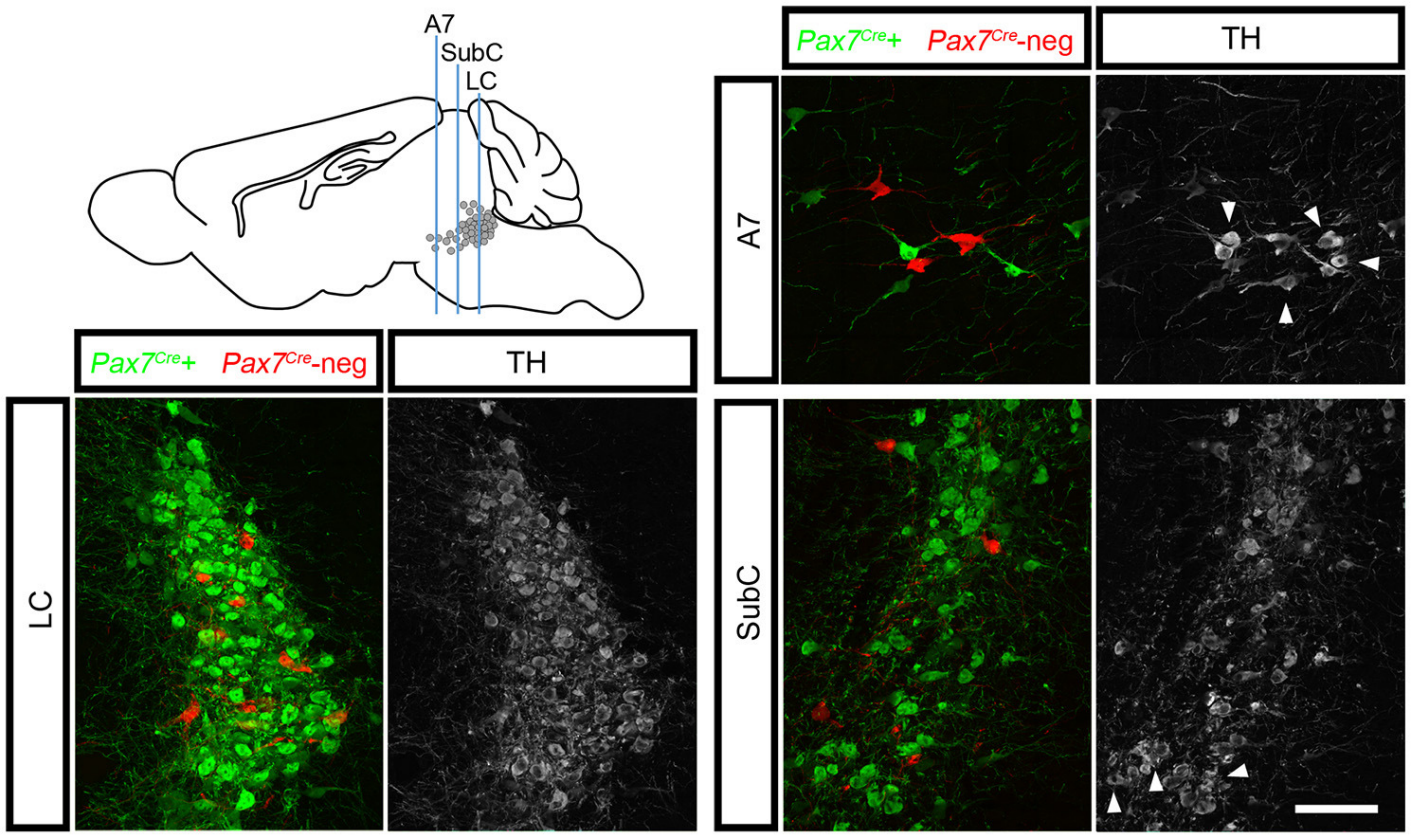
*Pax7*^*Cre*^-negative noradrenergic neurons are distributed throughout the LC complex. Sagittal schematic of the adult mouse brain shows approximate position of coronal sections from the brain of a 3-month old adult *En1*^*Dre*^*; Dbh*^*Flpo*^*; Pax7*^*Cre*^*; RC::RFLTG* quadruple heterozygote. In the locus coeruleus (LC), subcoeruleus (SubC), and A7 nuclei, *Pax7*^*Cre*^-negative noradrenergic neurons (tdTomato) are intermingled with *Pax7*^*Cre*^-positive noradrenergic neurons (EGFP). Anti-tyrosine hydroxylase (TH) immunostaining (gray) confirms the noradrenergic identify of labeled neurons. Noradrenergic neurons in SubC and A7 that originate outside *En1*^*Dre*^-defined r0 and r1 are TH-positive but are not labeled by the indicator allele (arrowheads). Scale bar: 100 μm.

To determine whether this subpopulation contributes to all three regions of the LC complex, we tracked r0- and r1-derived noradrenergic neurons into the adult brain (*n* = 10 mice) where the LC, SubC, and A7 noradrenergic nuclei can be readily identified. To confirm that fluorescent labeling was restricted to noradrenergic neurons, we immunolabeled with an antibody against tyrosine hydroxylase (TH). We consistently observed *Pax7*^*Cre*^-negative noradrenergic neurons (tdTomato+, TH+) intermingled with *Pax7*^*Cre*^-positive noradrenergic neurons (EGFP+, TH+) in all three regions of the LC complex (Figure 3). As expected, in *Pax7*^*Cre*^-negative controls heterozygous for *En1*^*Dre*^ and *Dbh*^*Flpo*^, we observed tdTomato in all r0- and r1-derived noradrenergic neurons, and no noradrenergic neurons were labeled in other controls. Thus, we have uncovered two distinct populations within the noradrenergic primordium of *En1*-defined r0 and r1 that contribute to the adult LC complex.

**Figure 4:**
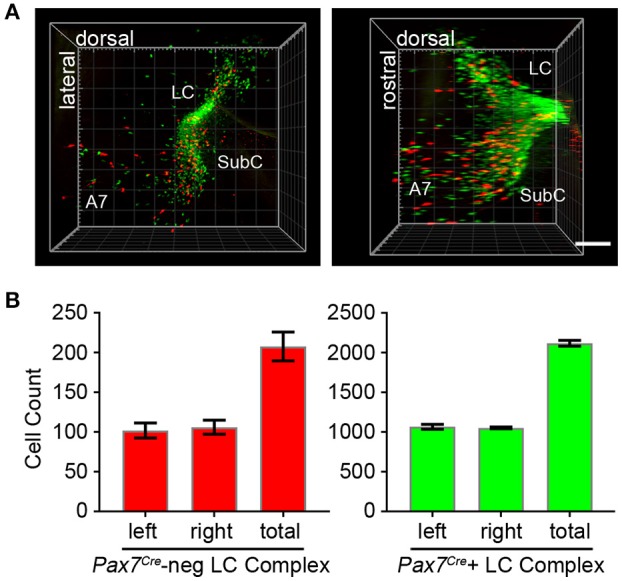
Quantification of *Pax7*^*Cre*^-negative and *Pax7*^*Cre*^-positive noradrenergic neurons in the LC complex. **(A)** Left LC complex from the brain of an adult *En1*^*Dre*^*; Dbh*^*Flpo*^*; Pax7*^*Cre*^*; RC::RFLTG* quadruple heterozygote cleared via the passive clarity technique (PACT) shows the spatial relationship of the r0- and r1-derived LC, SubC, and A7 nuclei. Immunolabeling of the intact tissue permits counting of *Pax7*^*Cre*^-negative (red) and *Pax7*^*Cre*^-positive (green) noradrenergic neurons throughout the LC complex. Left, view from anterior surface; Right panel, view from medial surface; scale bar, 300 μm. See also Supplementary Video 1. **(B)** Bilateral cell counts of *Pax7*^*Cre*^-negative and *Pax7*^*Cre*^-positive LC complex noradrenergic neurons from PACT-cleared brains of 4 to 5-month old mice.

To fully visualize the three-dimensional distribution of these two populations of noradrenergic neurons, we used the passive clarity technique (Yang et al., 2014) to clear adult brain tissue. Imaging and three-dimensional rendering of the intact LC complex (*n* = 6 mice) demonstrated that *Pax7*^*Cre*^-negative (tdTomato-labeled) and *Pax7*^*Cre*^-positive (EGFP-labeled) noradrenergic neurons are intermingled throughout the rostrocaudal and dorsoventral axes (Figure 4A and Supplementary Video 1). Next, we performed counts of tdTomato and EGFP-labeled neurons (*n* = 6 mice) to determine the relative contribution of the two subpopulations (Figure 4B). In the bilateral LC complex, we counted 2,326 ± 36 labeled neurons, a value intermediate between two previous estimates of cell numbers in the LC, including an undefined portion of the SubC, from C57BL/6 mice (Berger et al., 1979; Touret et al., 1982). Of that total, 208 ± 18 neurons were *Pax7*^*Cre*^-negative (tdTomato-labeled) and 2,118 ± 36 were *Pax7*^*Cre*^-positive (eGFP-labeled). Thus, the *Pax7*^*Cre*^-negative population constitutes 8.93 ± 0.74% of the full LC complex. Because the LC complex was imaged bilaterally, we were able to compare left and right sides. We observed no significant difference in the number of *Pax7*^*Cre*^-negative (*p* = 0.755, unpaired *t*-test) or *Pax7*^*Cre*^-positive neurons (*p* = 0.677, unpaired *t*-test).

**Figure 5:**
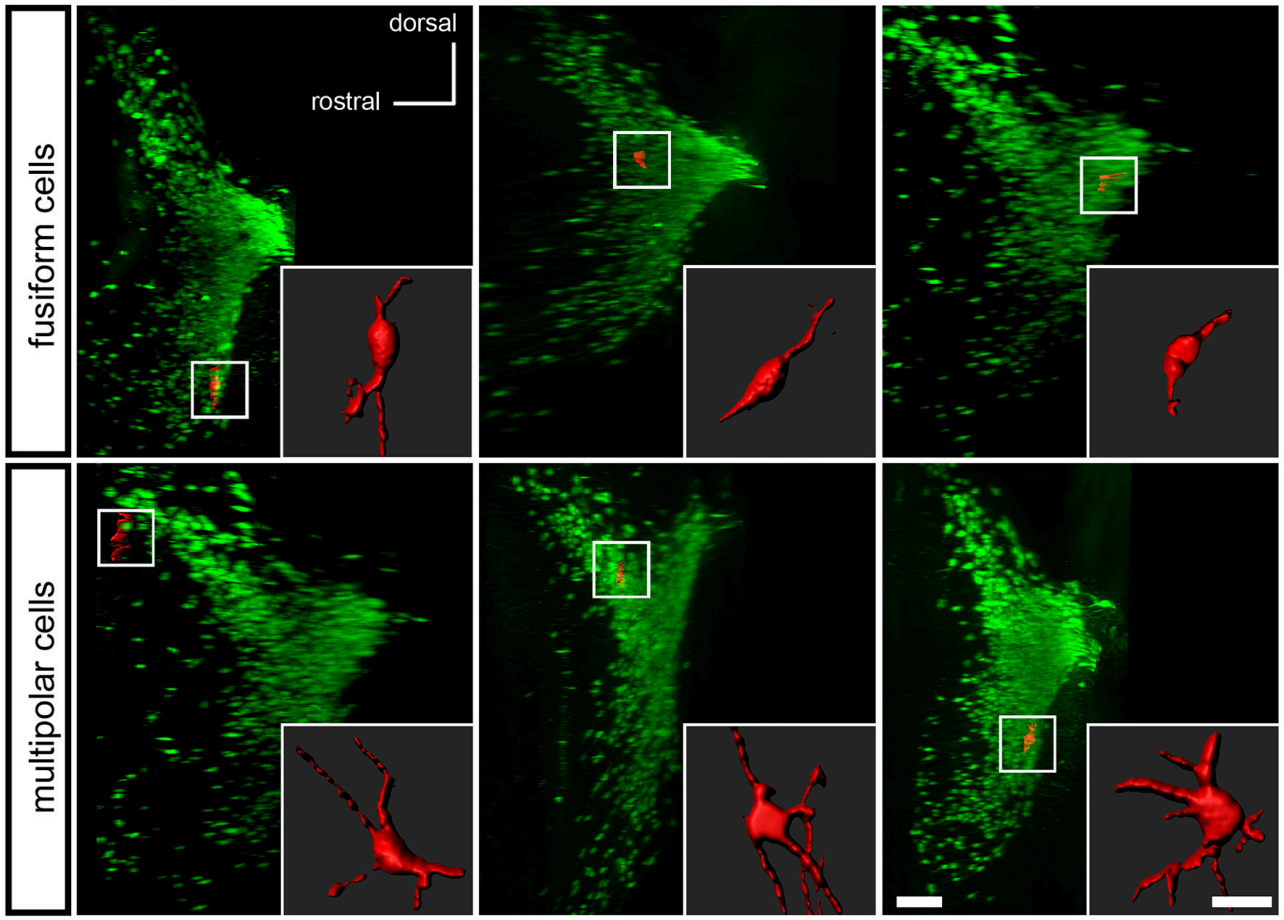
Morphology of *Pax7*^*Cre*^-negative noradrenergic neurons in the LC complex. In the PACT-cleared LC complex of 4–5-month old mice, the *Pax7*^*Cre*^-negative noradrenergic neurons are fusiform, generally with one or more processes at each end of the long axis **(top)**, or multipolar **(bottom)**. A surface rendering of each neuron shown in coronal view is inset on a sagittal view of the LC complex indicating the location of the neuron. Scale bar: 200 μm (LC), 40 μm (individual cells).

To determine if the *Pax7*^*Cre*^-negative population defines a morphologically distinct subset of LC complex neurons, we examined the tissue cleared by PACT. Studies of LC neuron morphology in the rat have previously indicated the existence of three main cell types: medium sized multipolar neurons, medium-sized fusiform neurons, and a few small ovoid neurons (Swanson, 1976; Shimizu et al., 1978; Cintra et al., 1982). Retrograde tracing and immunolabeling has demonstrated that both classes of medium-sized neurons are noradrenergic projection neurons (Loughlin et al., 1986). The identity of the small ovoid cells is uncertain, and different studies disagree as to whether they are noradrenergic (Shimizu et al., 1979; Loughlin et al., 1986). Because the tdTomato-labeled, *Pax7*^*Cre*^-negative neurons were dispersed in the LC complex, we could assess the shape of individual cells, similar to previous studies utilizing Golgi staining (Shimizu et al., 1978; Cintra et al., 1982). Roughly one quarter of the neurons examined were fusiform, and three quarters were multipolar (Figure 5). We did not observe any neurons that could be unequivocally assigned to the small ovoid category. Thus, the subpopulation of LC complex neurons originating outside the *Pax7*^*Cre*^ domain appears to include both morphologies of medium sized noradrenergic projection neurons.

**Figure 6:**
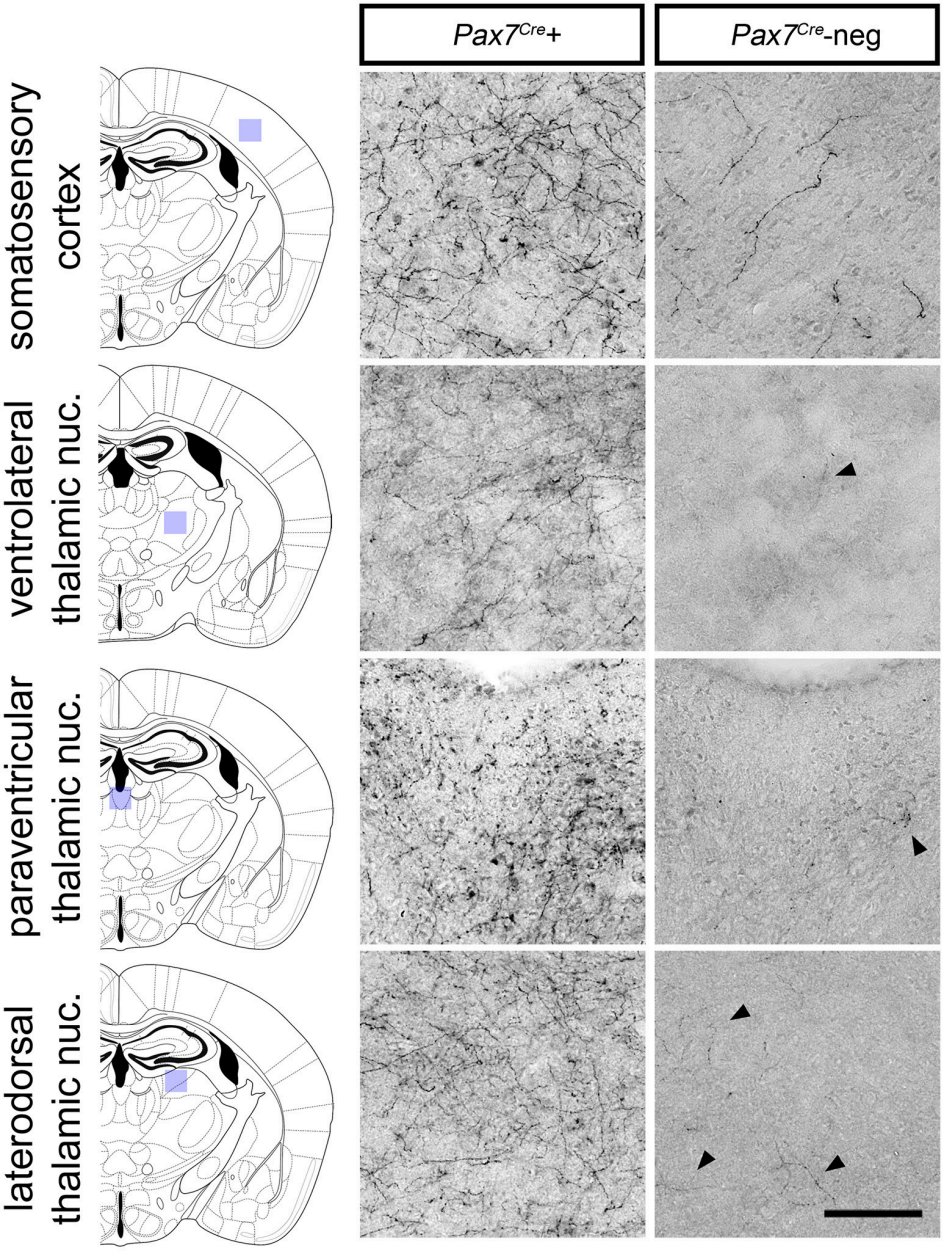
Axonal projections of *Pax7*^*Cre*^-negative and *Pax7*^*Cre*^-positive LC complex noradrenergic neurons. In representative coronal sections from the brain of a 3-month old adult *En1*^*Dre*^*; Dbh*^*Flpo*^*; Pax7*^*Cre*^*; RC::RFLTG* quadruple heterozygote, dense innervation by axons of *Pax7*^*Cre*^-positive (EGFP-labeled) neurons in the LC complex is observed in cortex and thalamus. Axons from the smaller *Pax7*^*Cre*^-negative (tdTomato-labeled) subpopulation are consistently observed in cortex, but they are very sparse in thalamus. Black arrowheads indicate occasional tdTomato-labeled axons in representative sections from thalamic nuclei. Scale bar: 100 μm.

Despite its relatively small size, this *Pax7*^*Cre*^-negative subpopulation is likely functionally significant if its axon projection profile differs from that of the larger *Pax7*^*Cre*^-positive subpopulation. To begin to address this question, we compared the axon projection profiles of the two subpopulations by exploiting the unique ability of the *RC::RFLTG* indicator allele to label axons of both the *Pax7*^*Cre*^-negative (tdTomato+) and *Pax7*^*Cre*^-positive (EGFP+) populations. For the most sensitive detection of axons, we labeled *Pax7*^*Cre*^-positive (EGFP+) and *Pax7*^*Cre*^-negative (tdTomato+) axons separately in sections from adult brain (*n* = 8 mice) using a horse radish peroxidase-linked secondary antibody and 3,3′-diaminobenzidine (DAB) staining. Axons from the LC complex project throughout the brain and provide the major source of norepinephrine to the cortex and thalamus (Robertson et al., 2013). Upon examination of sections labeled for the *Pax7*^*Cre*^-negative population, we observed a striking absence of axonal inputs to the thalamus (Figure 6). In contrast, inputs from the *Pax7*^*Cre*^-positive population were consistently present in thalamus, and we observed axons from both populations throughout the cortex, although there were fewer *Pax7*^*Cre*^-negative axons, as expected for a smaller population (Figure 6). This axonal projection pattern suggests that the *Pax7*^*Cre*^-negative neurons may be functionally distinct from the larger population of *Pax7*^*cre*^-positive LC complex neurons, highlighting the importance of early gene expression in generating differences in the neuronal circuitry.

## Discussion

Previous studies have clearly shown that neurons of the locus coeruleus are heterogeneous with respect to anteroposterior origin. Our initial intersectional fate mapping experiments demonstrated that the LC is primarily derived from the *En1* expression domain encompassing r0 and r1, with a very small subpopulation derived from r2 (Robertson et al., 2013). The more recent single-gene fate mapping using *Fgf8*^*Cre*^ showed that noradrenergic neurons derived from r0, as defined by *Fgf8* expression, contribute to the dorsal LC while those derived from r1 contribute to the ventral LC (Watson et al., 2017). Our results here reveal that the LC is also developmentally and genetically heterogeneous with respect to dorsoventral origin. Furthermore, the portions of the SubC and A7 nuclei included within the *En1*-derived LC complex are similarly composed of *Pax7*^*Cre*^-positive and *Pax7*^*Cre*^-negative subpopulations. The projection patterns of the two *Pax7*^*Cre*^-defined subpopulations of the LC complex may point to functional differences; the virtual absence of projections to the thalamus suggests that the *Pax7*^*Cre*^-negative subpopulation does not directly participate in the modulation of thalamic neuron activity performed by the broader LC complex (Berridge and Waterhouse, 2003). Taken together, these new data revealing (1) further heterogeneity within the anatomically defined LC, and (2) genetically defined subpopulations spanning several anatomically defined noradrenergic nuclei, offer further support for the idea that the anatomical divisions of the noradrenergic system are artificial constructs overlaid on natural genetic neuroanatomy arising from developmental history.

Limitations of the current study are the inability to overlay our *Pax7*^*Cre*^-defined noradrenergic fate map on the recently described *Fgf8*-defined subpopulations of the LC (Watson et al., 2017), and uncertainty regarding the precise dorsoventral origin of the *Pax7*^*Cre*^-negative subpopulation. We observe *Pax7*^*Cre*^-negative noradrenergic neurons throughout the dorsoventral extent of the adult LC complex, suggesting that they originate in both r0 and r1. However, it is possible that they originate in one domain and then migrate among the neurons derived from the other. A subpopulation as small as the *Pax7*^*Cre*^-negative noradrenergic neurons could easily be overlooked in single-gene fate-mapping experiments such as that which defined the dorsal r0-derived and ventral r1-derived LC subpopulations. Additional intersectional fate mapping experiments will be required to answer this question.

We are also unable to determine the precise dorsoventral level of the neural tube that is the source of the *Pax7*^*Cre*^-negative subpopulation of the LC complex, but the basal plate is a likely candidate. *Ascl1* (*Mash1*), a basic helix-loop-helix transcription factor required for noradrenergic neuron differentiation (Hirsch et al., 1998), is expressed in both alar and basal domains (Pattyn et al., 2004). It seems plausible that the basal *Ascl1* expression domain could give rise to a small population of noradrenergic neurons. Other possibilities include the floor plate, known to be the origin of dopaminergic neurons in the *En1*-expressing mesencephalon (Kittappa et al., 2007; Ono et al., 2007; Bonilla et al., 2008; Joksimovic et al., 2009; Blaess et al., 2011), or an as yet undetected alar microdomain which does not express *Pax7*^*Cre*^. We cannot fully rule out mosaic Cre expression within the alar *Pax7* expression domain as the source of the *Pax7*^*Cre*^-negative subpopulation, but the consistent size of the subpopulation and its distinct projection profile argue against this interpretation of the data. Efficient *Pax7*^*Cre*^-mediated recombination of a fluorescent Cre indicator that is a direct derivative of *RC::RFLTG* (Figure 1A) also indicates that the *Pax7*^*Cre*^-negative population is not an artifact of incomplete recombination. Regardless of origin, however, differential *Pax7*^*Cre*^ expression reveals two subpopulations of the LC complex that are molecularly distinct and experimentally separable.

Without a marker for the precise dorsoventral domain in which the *Pax7*^*Cre*^-negative subpopulation arises, its function cannot be experimentally assessed using genetic tools that depend on recombinase expression to drive intersectional expression of effector molecules such as hM3Dq (Sciolino et al., 2016). However, our ability to fluorescently label these two LC complex subpopulations would permit transcriptional profiling to identify differentially expressed genes, and subsequent generation of a recombinase driver expressed in the *Pax7*^*Cre*^-negative subpopulation. These future investigations promise to extend our knowledge of how embryonic cells with different developmental origins combine to give rise to the molecular and cellular heterogeneity of the mature LC complex. Such knowledge will be required for an understanding of how heterogeneity within the LC complex contributes to its regulation of diverse behaviors in the adult, and the roles that its dysfunction plays in neurological disease.

## Author contributions

PJ and NP designed and planned the experiments. PJ, NP, ES, KS, and CT performed the experiments. PJ, NP, KS, and ES analyzed the data. NP and ES prepared the figures. PJ and NP wrote the manuscript with input from co-authors.

### Conflict of interest statement

The authors declare that the research was conducted in the absence of any commercial or financial relationship that could be construed as a conflict of interest.

## Supplementary material

Supplementary Video 1 The intact structure of the LC complex and the spatial relationship of *Pax*^*Cre*^-positive and *Pax7*^*Cre*^ negative subpopulations are revealed in brain tissue cleared by the passive clarity techinque. After PACT clearing and immunostaining of *En1*^*Dre*^*; Dbh*^*Flpo*^*; Pax7*^*Cre*^*; RC::RFLTG* quadruple heterozygous brain, the noradrenergic neurons of the LC complex are observed to form a continuum. *Pax7*^*Cre*^-negative (tdTomato-labeled) neurons are intermingled with *Pax7*^*Cre*^-positive (EGFP-labeled) neurons throughout the dorsoventral and anteroposterior axes of the LC complex. At the beginning of the movie, the LC complex is viewed from the anterior, with the lateral surface to the left.
